# Correction to: Andexanet alfa in the treatment of acute major bleeding related to apixaban and rivaroxaban: a profile of its use in the USA

**DOI:** 10.1007/s40267-018-0576-1

**Published:** 2018-11-17

**Authors:** Young-A Heo

**Affiliations:** Springer, Private Bag 65901, Mairangi Bay, 0754 Auckland, New Zealand

## Correction to: Drugs & Therapy Perspectives (2018) 34(11):507–512 10.1007/s40267-018-0561-8

The article Andexanet alfa in the treatment of acute major bleeding related to apixaban and rivaroxaban: a profile of its use in the USA, written by Young-A Heo, was originally published Online First without open access. After publication in volume 34, issue 11, pages 507–512, Portola Pharmaceuticals, Inc. requested that the article be Open Choice to make the article an open access publication. Post-publication open access was funded by Portola Pharmaceuticals, Inc. The article is forthwith distributed under the terms of the Creative Commons Attribution-NonCommercial 4.0 International License (http://creativecommons.org/licenses/by-nc/4.0/), which permits any noncommercial use, duplication, adaptation, distribution and reproduction in any medium or format, as long as you give appropriate credit to the original author(s) and the source, provide a link to the Creative Commons license and indicate if changes were made.

The original article has been corrected.

